# pH and ROS Responsiveness of Polymersome Nanovaccines for Antigen and Adjuvant Codelivery: An In Vitro and In Vivo Comparison

**DOI:** 10.1021/acs.biomac.3c01235

**Published:** 2024-01-18

**Authors:** Eliézer Jäger, Olga Ilina, Yusuf Dölen, Michael Valente, Eric A.W. van Dinther, Alessandro Jäger, Carl G. Figdor, Martijn Verdoes

**Affiliations:** † Institute of Macromolecular Chemistry, Academy of Sciences of the Czech Republic, Heyrovsky Sq. 2, 162 06 Prague, Czech Republic; ‡ Department of Medical BioSciences, Radboud University Medical Center, Geert Grooteplein Zuid 28, 6525 GA Nijmegen, The Netherlands; § Institute for Chemical Immunology, Geert Grooteplein Zuid 28, 6525 GA Nijmegen, The Netherlands

## Abstract

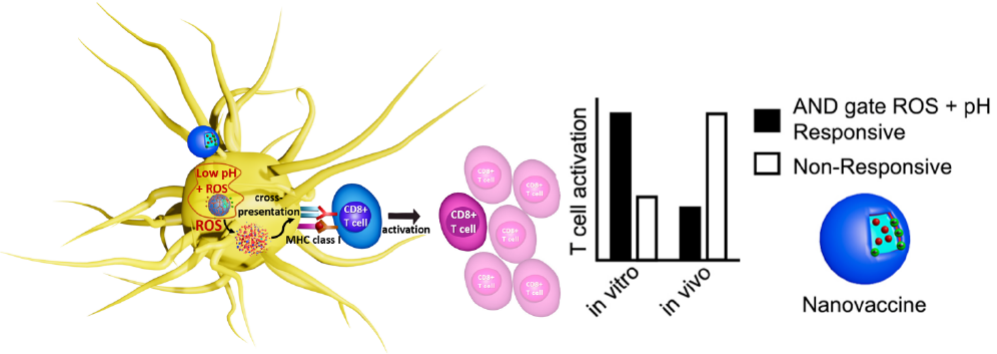

The antitumor immunity can be enhanced through the synchronized codelivery of antigens and immunostimulatory adjuvants to antigen-presenting cells, particularly dendritic cells (DCs), using nanovaccines (NVs). To study the influence of intracellular vaccine cargo release kinetics on the T cell activating capacities of DCs, we compared stimuli-responsive to nonresponsive polymersome NVs. To do so, we employed “AND gate” multiresponsive (MR) amphiphilic block copolymers that decompose only in response to the combination of chemical cues present in the environment of the intracellular compartments in antigen cross-presenting DCs: low pH and high reactive oxygen species (ROS) levels. After being unmasked by ROS, pH-responsive side chains are exposed and can undergo a charge shift within a relevant pH window of the intracellular compartments in antigen cross-presenting DCs. NVs containing the model antigen Ovalbumin (OVA) and the iNKT cell activating adjuvant α-Galactosylceramide (α-Galcer) were fabricated using microfluidics self-assembly. The MR NVs outperformed the nonresponsive NV in vitro, inducing enhanced classical- and cross-presentation of the OVA by DCs, effectively activating CD8+, CD4+ T cells, and iNKT cells. Interestingly, in vivo, the nonresponsive NVs outperformed the responsive vaccines. These differences in polymersome vaccine performance are likely linked to the kinetics of cargo release, highlighting the crucial chemical requirements for successful cancer nanovaccines.

## Introduction

In the past decades, cancer immunotherapy has become available as a treatment option in addition to conventional cancer treatments such as surgery, radiotherapy, and chemotherapy. Cancer immunotherapy uses engineered cells and synthetic or biological agents to initiate, modulate, and control an anticancer immune response. It attacks cancer via a different mechanism than chemo- and radiotherapy and thereby also holds the promise of being capable of destroying chemo-/radiotherapy-resistant tumors.^1^ Cancer vaccination is an example of cancer immunotherapy that aims to boost or induce a *de novo* adaptive immune response against tumor antigens. In this setting, it has been demonstrated that antitumor immunity can be enhanced by the synchronized delivery of antigens and immunostimulatory agents to antigen-presenting cells (APCs), particularly dendritic cells (DCs).^2,3^ An effective approach for synchronized codelivery is encapsulation of these vaccine components in biodegradable microparticulate- (MPs) or nanoparticulate (NPs) vaccine carriers (NVs).^4−8^ Indeed, it has been shown that NVs loaded with both the model tumor antigen ovalbumin (OVA) and the adjuvant α-galactosylceramide (α-Galcer) within the same vehicle induce immune responses that are more potent than those of either factor alone. α-Galcer serves as a remarkably potent agonist for invariant natural killer T-cell (iNKT) and functions as a DC transactivator. It amplifies antitumor immune responses by stimulating the secretion of diverse pro-inflammatory cytokines, thereby activating a spectrum of immune cells against the tumor.^9^

In order to activate T cells, the DCs have to internalize the antigen-containing NVs. After internalization, the antigen has to be released from the carrier to be processed into peptide epitopes, which are loaded onto major histocompatibility complex class II (MHCII) to be presented to CD4+ helper T cells or major histocompatibility complex class I (MHCI) for presentation to CD8^+^ cytotoxic T cells.^10,11^ The latter process is called cross-presentation. Both CD4^+^ and CD8^+^ T-cell responses are pivotal for successful cancer immunotherapy.^10−12^

Because vaccine cargo release after uptake is an essential step, NVs have been designed to be responsive to the inherent features of the DCs intracellular compartments.^13−15^ For instance, proposed “smart” NVs would be able to release their payloads triggered by, e.g., reduced pH, hypoxia, specific enzymatic activities, or reactive oxygen species (ROS). ROS plays a role in enhancing T-cell responses by promoting the maturation of APCs^16^ and cross-presentation.^16,17^ The ROS-responsiveness of NVs has been explored in several publications.^13,14^ For example, compared to nanocarriers containing noncleavable linkers, poly(propylene sulfide) (PPS) NPs with disulfide linkers release the antigenic cargo when the disulfide bond is cleaved in the reductive environment of the APC endosome, which leads to more robust CD8^+^ T-cell responses in vitro.^13^ In another approach, NVs that enhance the disruption of phagosomal compartments via the proton sponge effect^15^ were promising for the cytosolic delivery of tumor antigens in vivo to APCs in draining lymph nodes, generating strong cytotoxic CD8^+^ T-cell responses with low systemic cytokine expression.

However, coencapsulation of protein or peptide antigens together with an immunostimulatory adjuvant can be challenging due to the often very different physicochemical properties of the cargo components and the NV constituents (e.g., lipophilicity, charge, etc.).^3,18^ This challenge can be overcome by coencapsulating antigens and adjuvants in polymer vesicles, known as polymersomes (PSs).^19^ PSs are capable of compartmentalizing hydrophilic cargo in their aqueous lumen and hydrophobic cargo in their tunable membrane, rendering them very attractive for vaccine application.^19−21^ Compared to liposomes, PSs have enhanced stability without additional stabilization strategies such as cross-linking and are relatively more stable in blood circulation.^22^

Herein, we develop AND gate multiresponsive (MR) NVs containing the model antigen OVA and the adjuvant α-Galcer sensitive to two chemical cues encountered in DC endocytic compartments after NV uptake: low pH and high ROS levels. We hypothesized that the triggered release of antigen and adjuvant would result in enhanced antigen presentation by DCs and, thereby, more efficient activation of CD8^+^, CD4^+^ T cells, and iNKT cells, compared to the codelivery with nonresponsive NVs. The relative T cell activation efficacy of the responsive and nonresponsive NVs was assessed in vitro and in vivo.

## Experimental Section

### Materials and Chemicals

α-galactosylceramide (α-Galcer) was purchased from Cayman Chemical. Poly(*D*, *L*- lactide-*co*-glycolide (a mole ratio of 50:50, RESOMER RG 502 H, PLGA), Ovalbumin, Sephadex G50, Dulbecco’s phosphate buffered saline (PBS), dialysis kit Pur-A-Lyzer Maxi-6000 MWCO 6–8 kDa, and Amicon Ultra-4 Centrifugal filter unit were all purchased from Sigma-Aldrich. Ovalbumin Alexa-Fluor 488 and Ovalbumin Alexa-Fluor 647 were purchased from Thermo Fischer Scientific. Human IFN-γ uncoated ELISA and Mouse IL-2 Uncoated ELISA kits were purchased from Invitrogen. Solvents were purchased from Lachner and dried over molecular sieves (3 Å). The block copolymers poly[*N*-(2-hydroxypropyl)methacrylamide]-*b*-poly[*N*-(4-isopropylphenylacetamide)ethyl methacrylate] (PHPMA_25_-*b*-NR_33_, herein named NR block), poly[*N*-(2-hydroxypropyl)methacrylamide]-*b*-poly[*N*-(4-ethylamino]carbonyloxymethyl) phenylboronic acid pinacol ester methacrylate] (PHPMA_25_-*b*-MRE_30_, herein named MRE block), and poly[*N*-(2-hydroxypropyl)methacrylamide]-*b*-poly[*N*-(4-isopropylamino]carbonyloxymethyl) phenylboronic acid pinacol ester methacrylate] (PHPMA_25_-*b*-MRI_26_, herein named MRI block) were synthesized as previously described.^23^ The block copolymer poly[*N*-(2-hydroxypropyl)methacrylamide]-*b*-poly[4-(4,4,5,5-tetra-methyl-1,3,2-dioxaborolan-2-yl)benzyl methacrylate] (PHPMA_37_-*b*-ROS_42_, herein named ROS block) and the block copolymer poly([*N*-(2-hydroxypropyl)] methacrylamide)-*b*-poly[2-(diisopropylamino)ethyl methacrylate] (PHPMA_35_-*b*-PDPA_75_, herein named pH block) were synthesized according to our previously reported synthetic pathways.^24,25^ The subscripts refer to the degrees of polymerization of each block, as determined by ^1^H NMR. Table S1 reveals the polymer block physicochemical characteristics.

### Methods

#### Dynamic Light Scattering (DLS)

The Z-average diameter and the polydispersity index (PDI) were obtained from the autocorrelation function using the “general purpose mode” performed by using the Zetasizer NanoZS, Model ZEN3600 (Malvern Instruments, UK). The Dispersion Technology Software version 6.01 from Malvern was used to collect and analyze the data. One mL of the PSs (0.2 mg) was measured in polystyrene half-micro cuvettes (Fisher Emergo, Landsmeer, The Netherlands). The measurements were made at a position of 4.65 mm from the cuvette wall with an automatic attenuator and at a controlled temperature of 25 and 37 °C. For each sample, 1 run of 45 s were performed, with at least 5 repetitions for all the PSs.

#### Static Light Scattering (SLS)

For the SLS measurements, the scattering angle was varied from 30 to 150° with a 10° stepwise increase. The absolute light scattering is related to the weight-average molar mass [*M*_w(PSs)_] and radius of gyration (*R*_G_) of the PSs by the Zimm formalism, represented as
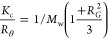
1where *K* represents the optical constant, encompassing the square of the refractive index increment (d*n*/d*c*); *R*_θ_ denotes the excess normalized scattered intensity (toluene was employed as the standard solvent); and *c* represents the polymer concentration given in mg·mL^–1^. The refractive index increment (d*n*/d*c*) of the PSs in pure water was determined using a Brice–Phoenix differential refractometer operating at a wavelength (λ) of 632.8 nm.

#### Electrophoretic Light Scattering (ELS)

The average zeta potential (ζ) of the PSs was measured using a Zetasizer Nano-ZS, Model ZEN3600 Instrument (Malvern Instruments, U.K.). The instrument measures electrophoretic mobility (*U_E_*) and converts the obtained value into ζ-potential (mV) using Henry’s equation (eq 2). In this equation, ε represents the dielectric constant of the medium, and *f*(ka) is Henry’s function calculated using the Smoluchowski approximation with *f*(ka) = 1.5 using the DTS (Nano) program.


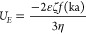
2

#### Cryogenic Transmission Electron Microscopy (Cryo-TEM)

Cryo-TEM investigations were conducted using a Tecnai G2 Spirit Twin 120 kV instrument (FEI, Czech Republic). A 4 μL solution of the NVs sample was loaded into an electron microscopy grid coated with a holey or lacey carbon supporting film (Electron Microscopy Science), hydrophilized through glow discharge (Expanded Plasma Cleaner, Harrick Plasma) just before the experiment. Excess solution was removed by blotting (Whatman no. 1 filter paper) for approximately 1 s, and the grid was plunged into liquid ethane maintained at −182 °C. The vitrified sample was promptly transferred to the microscope and observed at −173 °C at an accelerating voltage of 120 kV.

#### Manufacture of the Nanovaccines

The PSs were manufactured after testing diverse polymer concentrations, organic solvents, and flow-rates for the organic (OP) and aqueous phases (WP), in accordance with the previously described method.^23−25^ Briefly, for 1:1 ratio OP/WP, the BCs (1 mg·mL^–1^) were dissolved in THF/MeOH (80/20) (v/v) containing α-Galcer (5 μg), and OVA (50 μg) was dissolved in PBS pH 7.4 (1 mL). PSs were produced using the microfluidic device setup from Dolomite (Royston, United Kingdom) equipped with a glass Micromixer chip with 12 mixing stages microchannels of 50 μm × 125 μm (depth × width). The polymer solution was pumping through the middle channel and PBS solution (pH 7.4) through the side channels using two independent Dolomite Mitos P-Pump (Royston, United Kingdom) controlled via PC software. All of the solutions were previously filtrated (0.22 μm, Millipore). The obtained PSs were passed through a Sephadex G50 column in PBS (pH 7.4) to remove organic solvents and none encapsulated OVA and α-Galcer and collected by using a UV–vis detector. The PSs were concentrated to 1 mL by using an Amicon Ultra-4 Centrifugal filter unit.

PLGA nanoparticles encapsulating ovalbumin and α-Galcer were prepared using a single emulsion and solvent evaporation-extraction method as described previously.^8^ Briefly, 100 mg of PLGA in 3 mL of dichloromethane containing 5 mg of OVA and 100 μg of α-Galcer dissolved in DMSO were added to 25 mL of aqueous phase containing 2% poly(vinyl alcohol) and emulsified for 120 s using a digital probe sonicator (Branson Ultrasonics, Danbury, CT). The organic phase was evaporated overnight at 4 °C, and the PLGA nanovaccines were collected by centrifugation at 14 000 rpm for 20 min, washed six times with ultrapure water, and lyophilized. OVA and α-Galcer contents were determined as described below.

The OVA loading content (LC) and the OVA loading efficiency (LE) were calculated using the standards equations:


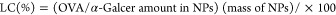
3


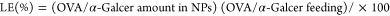
4

OVA/α-Galcer-unloaded PSs (empty PSs) were prepared by the aforementioned procedure, however, without the addition of the antigen and adjuvant.

#### Antigen and Adjuvant Content

The ovalbumin content of the NVs was assessed using a Coomassie Plus Protein Assay Reagent (Pierce) following the manufacturer’s protocol. The α-Galcer content of the NVs was determined through a Corona Veo charged aerosol detector (CAD) connected to an UltiMate 3000 high-performance liquid chromatography (HPLC) system (Thermo Fischer Scientific) following previously published protocol.^26^ The NVs were dissolved in DMSO to ensure complete dissolution of the components and were subsequently analyzed by CAD using an XSelect CSH C18 2.5 μm 3.0 × 150 mm XP column (Waters), eluents H_2_O-ACN-MeOH with ACN-MeOH gradient 0–100 vol %, flow rate = 1.0 mL·min^–1^. The quantity of α-Galcer was determined through interpolation using standard calibration curves of α-Galcer, prepared in the same manner as that for the NVs.

#### Antigen Release Experiments

The OVA-Alexa 647 in vitro release experiments were performed by dialysis method following previously published protocol^27^ in three different conditions: PBS (pH 7.4), PBS with 1 mM H_2_O_2_ at pH 5.3 (acetate buffer), and at 1 mM H_2_O_2_. A preswollen cellulose dialysis membrane tube with a molecular weight cutoff (MWCO) of 6–8 kDa (Pur-A-Lyzer) was filled with 2.0 mL of OVA-Alx647-loaded-PSs at a concentration of 5 μg·mL^–1^. The membrane tube was then immersed in 3 L of the specific buffer aforementioned at 37 °C and 350 rpm. At predetermined intervals, 10 μL of the PSs was sampled from the interior of the dialysis tubing, and the absorbance of OVA-alx647 was measured using a NanoDrop 2000 spectrophotometer at 650 nm. Following this, the sampled solution was returned to the respective membrane tube.

#### Enzyme-Linked Immunosorbent Assays (ELISAs)

ELISAs were utilized for the quantitative detection of human interferon γ (IFN-γ) and mouse interleukin-2 (IL-2) produced during the incubation of the DC-T-cell cocultures. Human IFN-γ uncoated ELISA and mouse IL-2 uncoated ELISA kits (Invitrogen, Waltham, MA, USA) were employed following the manufacturer’s protocol. Serum samples for IFN-γ and IL-2 analysis were diluted 1/5 and 1/30, respectively, in blocking buffer before being added to the ELISA plates.

#### Cell Culture

JAWS II cell, a DC line, was used as a model cell for the uptake and first experiments. The JAWS II cells were cultured in MEM alpha medium with ribonucleosides, deoxyribonucleosides, l-glutamine (4 mM), sodium pyruvate (1 mM), murine GM-CSF (5 ng·mL^–1^), and fetal bovine serum (20% v/v) in Petridish (Greiner, 10 cm, 633185) at culture medium (10 mL) at 37 °C with 5% CO_2_.

DN32.D3 NKT cell hybridomas were cultured in full RPMI MEM alpha medium with ribonucleosides, deoxyribonucleosides, l-glutamine (4 mM), sodium pyruvate (1 mM), murine GM-CSF (5 ng/mL), and fetal bovine serum (20% v/v) in Petridish (Greiner, 10 cm, 633185) at culture medium (10 mL) at 37 °C with 5% CO_2_.

#### Culture of Dendritic Cells from Mouse Bone Marrow Cells

The murine DCs were prepared by following the reported protocol.^27^ C57BL/6 mice were sacrificed, and their femurs and tibia were harvested. The bone marrow was flushed out with culture medium (1640 RPMI medium plus 10% FCS, 1% glutamine, 1% Antibiotic-Antimycotic, and 50 μM 2-Mercaptoethanol) and collected by centrifuge at 1500 rpm for 5 min. For GM-CSF BMDCs preparation, the obtained bone marrow cells (4 × 10^6^) were plated out in Petridish (Greiner, 10 cm, 633185) with culture medium (13 mL) containing granulocyte–macrophage colony-stimulating factor (GM-CSF, Peprotech, 20 ng·mL^–1^), and cultured at 37 °C with 5% CO_2_ for 4 days. Afterward, fresh culture medium (4 mL) containing GM-CSF (37.2 ng·mL^–1^) was added, and the cells were cultured for another 3 days. The resulting nonadherent cells (day 6) were harvested and resuspended in fresh culture medium containing GM-CSF (8.75 ng·mL^–1^) for further DC maturation assays.

For CD103^+^ DCs preparation, the obtained bone marrow cells (15 × 10^6^ per 10 mL) were plated out in Petridish (Greiner, 10 cm, 633185) with culture medium (1640 RPMI medium plus 10% FCS and 1% of 2-Mercaptoethanol) containing fetal liver tyrosine kinase 3-Ligand (Flt3-L, 200 ng·mL^–1^) and GM-CSF (5 ng·mL^–1^), and cultured at 37 °C with 10% CO_2_ for 5 days. Subsequently, the cells were supplemented with complete medium (5 mL of culture medium containing Flt3-L and GM-CSF) and further cultured for 4 days. After that, the nonadherent cells were harvested, counted, and replated at a concentration of 3 × 10^6^ cells in complete medium (10 mL) and cultured for more 6 days. The resulting DCs (day 14) were used for further DC maturation assays and in vitro T cell activation assays.

#### In Vitro Activation of OT-I T Cells and DN32.D3 Cells

CD8+ T cells obtained from OT-I transgenic mice, referred to as OT-I T cells, were isolated via negative selection using CD8+ T-cell isolation kit II (Miltenyi) following the manufacturer’s protocol. The isolated cells were subsequently stained with CFSE (Thermo Fisher, 2.5 × 10^–6^ M) and used directly without the need for additional culture.

For the in vitro activation of OT-I T cells, GM-CSF cells or CD103+ BMDCs (10^4^ cells per sample) obtained on day 14 were incubated with various vaccines at different concentrations at 37 °C with 5% CO_2_ for 24 h. Subsequently, CFSE-labeled OT-I T cells (5 × 10^4^) were added and cocultured for 72 h. The proliferation of OT-I T cells was evaluated by using flow cytometry (BD FACSCalibur). The mean cycle was determined following the protocol described by Valente et al.^28^ Generally, the CFSE dilution factor (*f*) was computed by dividing the stimulated mean fluorescence intensity (MFI) by the unstimulated MFI. The mean cycle was then calculated by using the formula of “log2 (*f*).” In addition, the secretion of two critical cytokines, IFN-γ and IL-2, were analyzed through ELISA (Mabtech).

For the activation of DN32.D3 NKT cell hybridoma’s in vitro, CD103+ BMDCs (5 × 10^4^ cells per sample) obtained on day 14 were incubated with the OVA-αGalcer nanovaccines soluble controls (OVA+αGalcer) at various concentrations at 37 °C with 5% CO_2_ for 3 h. Following this, DN32.D3 cells (1 × 10^5^ cells) were introduced and cocultured for 24 h. The supernatant was collected for IL-2 ELISAs.

#### Mice

Wild-type C57BL/6JRccHsd (Harlan) and OT-I C57BL/6-Tg(TcraTcrb)1100Mjb/Crl (Charles River) mice were housed under specific pathogen-free conditions at the Central Animal Laboratory (Nijmegen, The Netherlands). They had ad libitum access to drinking water and food. All experiments were conducted in accordance with the guidelines for animal care set forth by the Nijmegen Animal Experiments Committee, adhering to the ethical standards described in the Declaration of Helsinki.

#### In Vivo Activation of OT-I T Cells

Celltrace violet (Life technologies)-labeled OT-I T cells (3 × 10^6^) were adoptively transferred into C57BL/6 mice by intravenous injection. The day after, mice were vaccinated with the different NVs at a dose of OVA corresponding to 0.04 and 0.01 μg. Three days later, the mice were sacrificed, and spleens were harvested. After organ mechanical disruption, splenocyte suspension was obtained, and cells were passed through 100 μm cell strainer (Falcon). The OT-I T cells were labeled with a FITC-tagged CD8 antibody (BD Biosciences). Proliferations of OT-I T cells were evaluated by dilution of Celltrace violet intensity measured with flow cytometry (FACS verse BD Biosciences). Fixable ViabilityDye eFluor 780 (ebioscience) or zombie violet (Biolegend) dyes were used to exclude dead cell in flow cytometry applications.

## Results and Discussion

**Figure 1:**
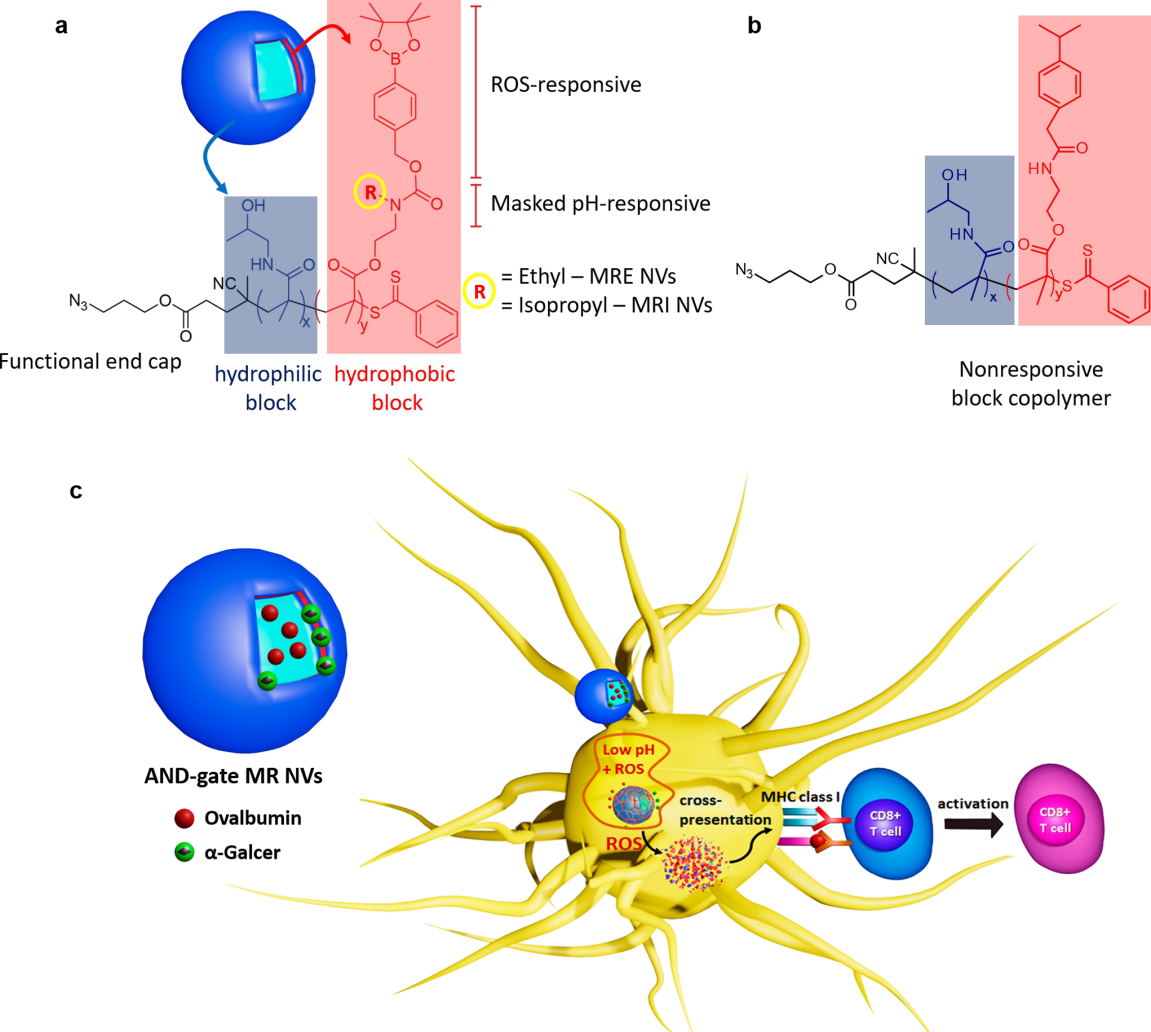
Schematic representation of the “AND gate” multiresponsive polymersome NVs approach. a) Structure of the “AND gate” multiresponsive diblock copolymers, composed of an alkyl-azide-capped (in black) PHPMA hydrophilic block (in blue), and a ROS-activatable pH-responsive hydrophobic block (in red). b) Structure of the side chain of the hydrophobic block of the nonresponsive block copolymer (in red). c) Working principle of the MR NVs for enhanced cytotoxic CD8^+^ T cell activation. NVs are first endocytosed by DCs and then evaded into cytosol because the higher ROS and low-pH conditions at the endosomes trigger NVs disassembly—antigens/adjuvants released, enhancing their cross-presentation, which is expected to enhance cytotoxic T cell activation.

We recently reported the development of functionalizable ROS and pH “AND gate” multiresponsive amphiphilic block copolymers (Figure 1a).^23^ The hydrophobic blocks contain 4-(hydroxymethyl)phenylboronic acid pinacol ester carbamate masked pH-responsive side chains, which are exposed exclusively in response to ROS (Figure 1a, red). Ethyl (MRE) and isopropyl (MRI) secondary amine side chains were synthesized as two versions of the pH-responsive entities, differing in charge-reversal pH and hydrophobicity (p*K*_a_ ∼ 5.8 for MRE; p*K*_a_ ∼ 5.5 for MRI), as well as a nonresponsive side-chain version (Figure 1b, red). These blocks are linked to an alkyl-azide-capped hydrophilic poly([*N*-(2-hydroxypropyl)]methacrylamide) (PHPMA) block (Figure 1a,b, blue). The optimized conditions for manufacturing the block copolymers were established based on our previously published protocols,^23−25^ with the molecular weights of the blocks set within the range of 10–25 kDa and low dispersity. The obtained molecular weight, hydrophilic/hydrophobic weight ratios of the dBCs (ϕ = volume fraction of the hydrophilic block = 10–40%) (Table S1) facilitate the preparation of well-defined polymersomes.^23−25^ We herein applied these polymer designs to generate polymersome NVs for the codelivery of OVA and α-Galcer and evaluated their in vitro and in vivo ability to induce antigen-specific T-cell activation (Figure 1c).

**Figure 2:**
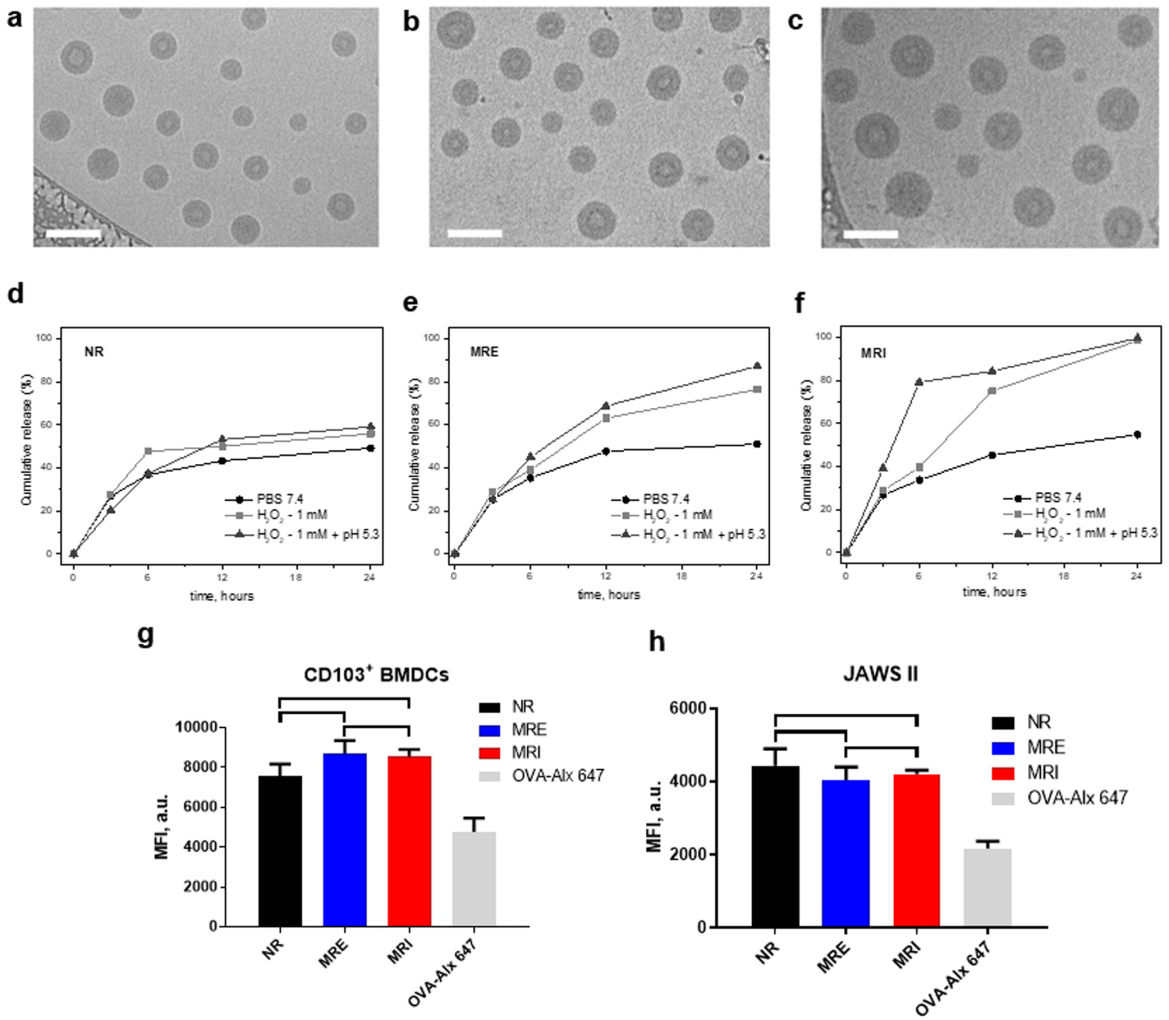
Cryo-TEM micrographs of the NR (a), MRE (b), and MRI (c) NVs (scale bar = 100 nm). Model antigen release (OVA-Alx647) from NR (d) and MRE (e) and MRI (f) NVs in PBS pH ∼ 7.4 (black circles), in the presence of 1 mM H_2_O_2_ pH ∼ 6.5 (light gray squares) and in the presence of 1 mM H_2_O_2_ pH ∼ 5.3 (dark gray triangles) along 24 h incubation at 37 °C. Cellular uptake of NVs after 3 h incubation in CD 103^+^ BMDCs (g) and JAWS II (h). One-way ANOVA, *p* < 0.05; only nonsignificant are depicted for clarity.

We utilized the hydrodynamic flow-focus nanoprecipitation microfluidic self-assembly protocol to assemble the block copolymers (BCs) into MR NVs with good reasonable control over antigen and adjuvant entrapment.^23−25^ The BCs (concentration ∼1 mg·mL^–1^) dissolved in THF/methanol (80/20) containing α-Galcer (5 μg·mL^–1^) as an organic phase and a phosphate buffer saline (PBS, pH ∼ 7.4) solution containing the model antigen OVA (50 μg·mL^–1^) as an aqueous phase were assembled in the microfluidics chip (see Methods for description). Spherical and uniform NVs were obtained as determined by cryo-transmission electron microscopy (cryo-TEM, Figure 2a–c) and dynamic light scattering (DLS, Table S1). The diameters as observed by cryo-TEM were confirmed by DLS with the distribution of diameters for the MR and NR NVs appearing as one single population with an average diameter of ∼120 nm (PdI = 0.116) for MRE, ∼ 134 nm (PdI = 0.115) for MRI, and of ∼124 nm (PdI = 0.119) for NR. These diameters are within a range known to be ideal for efficient DC uptake and antigen presentation.^8,29^

We have previously demonstrated the responsiveness of the MR polymers in the context of doxorubicin-loaded polymersomes to low pH (acetate buffer pH 5.3), ROS (1 × 10^–3^ M H_2_O_2_ pH ∼ 6.5), and the combination thereof (1 × 10^–3^ M H_2_O_2_ pH ∼ 5.3).^23^ The diameters of all polymersomes remained unchanged after 24 h of incubation in PBS 7.4 or at pH 5.3, demonstrating high stability under these conditions and unresponsiveness to acidity as a single chemical cue. Upon exposure to H_2_O_2_ at low pH, the diameters of the NR NVs remain unchanged. In contrast, a dramatic decrease in size is observed for the MRE and MRI demonstrating the AND gate dual-responsiveness of the MR polymersomes. To determine the responsiveness of the OVA and α-Galcer loaded polymersome NVs, the ROS- and pH-triggered cargo release was studied using Alexa-Fluor 647 labeled OVA (OVA-Alx647). The antigen release kinetics of the OVA-loaded MR and NR NVs were examined with fluorescence spectroscopy measurements over 24 h using the same conditions detailed above (Figure 2d–f). Under DC endosomal simulated conditions (H_2_O_2_ at low pH), the release of the OVA from the MR NVs was drastically enhanced compared to that of the NR counterpart. Moreover, the antigen release kinetics differ slightly between MRI and MRE, with MRI being more differentially responsive toward ROS at pH ∼ 6.5 or pH ∼ 5.3 (Figure 2f). The observed faster ROS and pH-dependent antigen release of the MR NVs are hypothesized to result in an antigen and adjuvant burst release after uptake by and subsequent endosomal maturation in the DC, which could impact the amplitude of antigen presentation and resulting T cell activation. Using flow cytometry, we next evaluated the cellular uptake of the MR and NR NVs loaded with OVA-Alx647 by JAWS II cells (an immortalized immature mouse DC cell line) and primary mouse CD103^+^ bone marrow-derived DCs (BMDCs).^8,27^ It is important to determine whether the different NVs are taken up to a similar extent by DCs when aiming to study the effects of vaccine cargo release by MR and NR NVs. All NVs were taken up to a similar extent by both DC types, resulting in more antigen uptake compared to exposure to free OVA-Alx647. Taking into consideration that the particle uptake is generally dependent on NP size, shape, and charge,^30,31^ the similar uptake behavior for the NVs was expected because their surface chemistry is similar (PHPMA shell), they are spherical in shape with similar diameters (PBS pH 7.4) being slightly negative in charged (ζ ≈ −5.6 ± 0.9 mV for NR, −6.1 ± 0.7 mV for MRE, and −2.0 ± 0.7 mV for the MRI) (Table S1).

**Figure 3:**
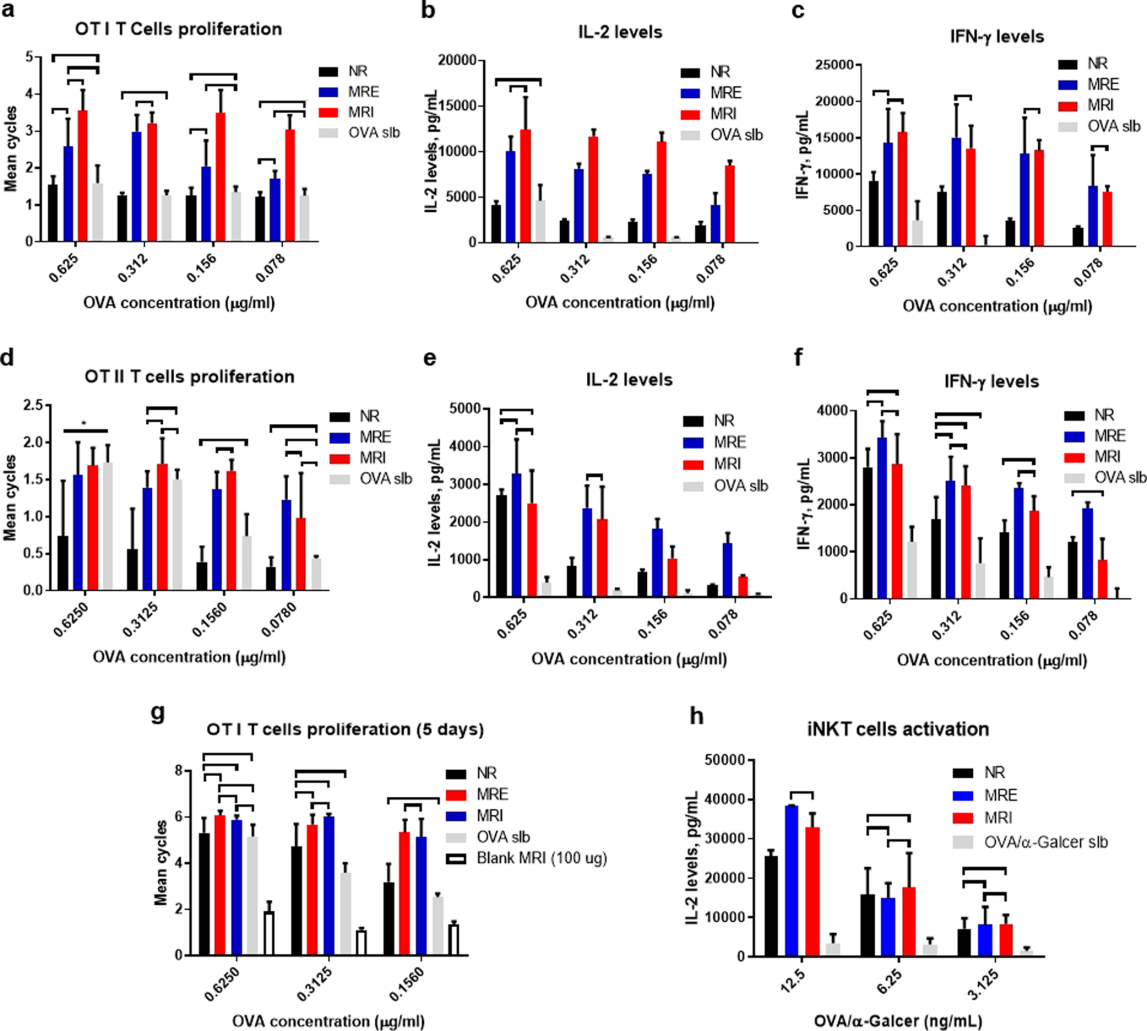
a) In vitro cross-presentation of the ovum-like OVA by DCs for priming OT-I T cells. Mean cell division cycles from the proliferation assay of CFSE-labeled OT-I T cells (a) after 72 h incubation with GM-CSF BMDCs, which were pretreated with soluble OVA (OVA slb) or NVs for 24 h. Corresponding IL-2 (b) and IFN-γ (c) levels. In vitro classical presentation of OVA by DCs for priming OT-II T cells. Mean cell division cycles from the proliferation assay of CFSE-labeled OT-II T cells (d) after 72 h incubation with GM-CSF BMDCs, which were pretreated with soluble OVA or NVs for 24 h. Corresponding IL-2 (e) and IFN-γ (f) levels. In vitro cross-presentation of the ovum-like OVA by DCs for priming OT-I T cells. Mean cell division cycles from the proliferation assay of CFSE-labeled OT-I T after 5 days of incubation with CD103^+^ BMDCs which were pretreated with soluble OVA or NVs for 24 h (g). IL-2 response curves of DN32.D3 NKT cell hybridoma cultured 24 h with CD103^+^ BMDCs which were pretreated with soluble OVA/α-Galcer (OVA/α-Galcer slb) or with the loaded-NR and MR NVs (h). One-way ANOVA, *p* < 0.05; only nonsignificant are depicted for clarity.

We next evaluated the capability of the NVs to enhance the cross-presentation of antigens by DCs to efficiently prime CD8+ T cells efficiently. Immature GM-CSF BMDCs were incubated with different NVs for 24 h. OVA-specific CD8^+^ T cells harvested from OT-I transgenic mice (termed OT-I T cells) were labeled with carboxyfluorescein diacetate succinimidyl ester (CFSE) and subsequently cocultured with the NR and MR NVs-treated BMDCs for 72h. CFSE dilution was measured by flow cytometry as a measure of OT-I T-cell proliferation.^8,27^ The average number of cell divisions^27,28^ was calculated and is depicted in Figure 3a.

The NVs chemistry strongly influenced the activation of CD8^+^ T cells in vitro. After three-day coculture, the OT-I T cell activation by DCs treated with MR NVs is superior over NR NVs and soluble OVA antigen for all concentrations evaluated, with MRI NVs being more potent than MRE NVs (Figure 3a, red columns). Similar trends are observed for IL-2 and IFN-γ production (Figure 3b,c), which could be attributed to the enhanced AND gate responsiveness of MRI as observed in the OVA release assay (Figure 2e,f). After 5 days of coculture, the OT-I T cell proliferation induced by the MR NVs is on par, while the NR NVs-treated DCs are also capable of inducing CD8^+^ T cell activation after a prolonged period, albeit less potent than the MR NVs (Figure 3g). These differences between 3- and 5-day cultures are likely explained by the increase in cumulative vaccine cargo release with longer incubation time. As expected, only background OT-I T cell activity is observed in the empty NVs condition, which is increased when the cognate antigen is encapsulated. Collectively, these results demonstrated that the MR NVs dramatically increase the CD8^+^ T cell priming efficiency of DCs compared to that of soluble antigens and NR NVs.

In parallel, we set out to investigate if DCs exposed to the NVs were capable of priming CD4^+^ T cells from OT-II mice (named OT-II T cells), which recognize a specific OVA-derived MHC II epitope (Figure 3d–f). Coculture of the NV-treated DCs induced OT-II cell proliferation (Figure 3d) and the production of IL-2 (Figure 3e) and IFN-γ (Figure 3f) in an antigen concentration-dependent manner. Superior CD4^+^ T cell activation is observed for the MR NVs compared to the NR NVs, with a seemingly more potent induction of cytokine production by the MRE NVs (Figure 3d).

Additionally, BMDCs incubated with the OVA/α-Galcer polymersome NVs were able to activate iNKT cells more potently compared to soluble OVA/α-Galcer as judged by the DN32.D3 NKT cell hybridoma activation assay,^8,27^ indicating increased α-Galcer presentation by CD1d (Figure 3h). Overall, the MR NVs-pulsed DCs can induce robust CD4 T cell, CD8 T cell, and iNKT cell activation, even at low OVA and α-Galcer concentrations, outperforming the free OVA and the NR NVs.

**Figure 4:**
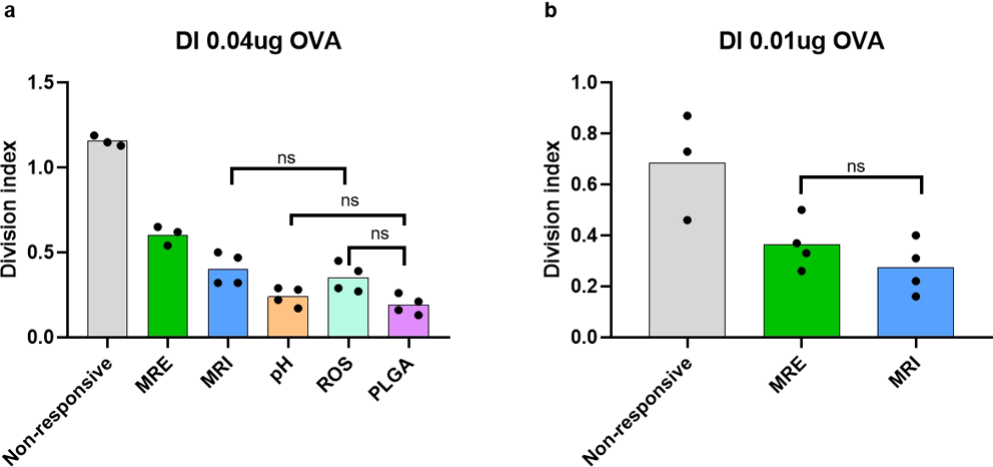
In vivo NV induced the cross-priming of OT-I T cells. The diagram shows the division index (average number of cell divisions that the responding cells underwent) of the CD8+ OT-I cell population of splenocytes of mice 72 h after intravenous injection with the different vaccines at 40 ng OVA (a) and 10 ng OVA (b). Representative of 3–4 mice per group. One-way ANOVA, *p* < 0.05; only nonsignificant are depicted for clarity.

Ultimately, the capacity of the NVs to induce the in vivo cross-priming of CD8^+^ T cells was tested. Celltrace violet-labeled OT-I T cells were transferred into naïve C57Bl/6 mice. One day later, the mice were vaccinated with equivalent amounts of OVA and α-Galcer loaded NR, MRE, and MRI NVs by intravenous injection at two doses (Figure 4a,b). To compare the performance of our polymersome NVs to an intravenously injected NV platform currently in clinical trial,^32^ mice were treated with OVA and α-Galcer loaded poly(lactic-*co*-glycolic acid) (PLGA) NV.^8^ To further assess the influence of the type of chemical responsiveness of the NVs on in vivo performance, we took along “single” pH-responsive NVs manufactured from the poly([*N*-(2-hydroxypropyl)] methacrylamide)_35_-*b*-poly[2-(diisopropylamino)ethyl methacrylate]_75_ block copolymer,^33^ as well as single ROS-responsive NVs made up of poly([*N*-(2-hydroxypropyl)] methacrylamide)_37_-*b*-poly[4-(4,4,5,5-tetra-methyl-1,3,2-dioxaborolan-2-yl)benzyl methacrylate)_42_^25^ block copolymer. Three days after vaccination, mice were sacrificed, spleens were harvested, and OT-I T cell proliferation was assessed by flow cytometry.

As shown in Figure 4, all tested NVs were able to induce OT-I T cell proliferation. Interestingly, the NR polymersome NVs outperformed the multiresponsive NVs, single responsive NVs, and the NVs prepared from PLGA. Overall, these results confirm the capability of these polymersome NVs, in particular the nonresponsive, to efficiently induce activation of antigen-specific cytotoxic CD8+ T cells in vivo.

Given that the PSs share similar characteristics such as size (∼130 nm in diameter), hydrophilic shell (PHPMA), surface charge (−2 to −6 mV), cargo content (40 ng of OVA, ESI), and in vitro uptake by DCs (Figure 2j,k), we hypothesize that the observed differences in in vivo performance of the tested PSs vaccines are not likely to be a result of, for example, differential immune cell type uptake, by rather linked to the kinetics of cargo release. The in vivo comparison of T-cell priming between stimuli-responsive and nonresponsive NVs after intravenous injection is limited in the literature, while it has been demonstrated that intravenous injection of OVA and iNKT cell agonist loaded NVs results in a more potent T cell activation compared to other injection routes.^34^ An inherent risk of using environmentally responsive NVs is the potential compromise of in vivo stability in circulation or in specific tissue environments. This could result in premature antigen and/or adjuvant release before DC uptake, which reduces the degree of synchronized codelivery of antigen and adjuvant.^35,36^ This observation was reported by Zhao et al.,^35^ evaluating the adjuvant release (R-848/MPLA) from PLGA NVs prepared from PLGA polymer with different inherent viscosities to obtain different antigen release kinetics. Because of the distinct release kinetics prior to being captured by antigen-presenting cells, a significant amount of adjuvant could be prematurely released from NVs that exhibit a rapid release rate. Conversely, for sustained-release NVs, less adjuvant is prematurely released, thereby ensuring that a greater amount of adjuvant remains available to antigen-presenting cells. Furthermore, Demento et al.^36^ emphasized the significance of sustained OVA release mediated by NVs prepared from PLGA, as opposed to liposomes. The faster release rate of OVA from the liposomes after subcutaneous injection resulted in less effective generation of effector-like CD8+ T cells in vivo. Therefore, the relative stability of the NR NVs in combination with the observed sustained antigen release in vitro could explain the enhanced performance of the NR NV over the stimuli-responsive NVs in vivo.

## Conclusion

In summary, we demonstrated that the model antigen OVA and iNKT cell activating adjuvant α-Galcer loaded polymersome NVs can induce robust in vitro and in vivo antigen-specific T cell activation. The “AND gate” ROS- and pH-responsiveness of the NVs enhances the in vitro classical antigen-presentation (MHCII) and cross-presentation (MHCI) kinetics and potency, making this platform an interesting approach in the context of ex vivo DC activation for DC vaccine cell-based therapy applications.^37,38^ The in vivo efficacy of the polymersome NVs outperforms the PLGA-based NV platform currently in phase-I clinical trial.^32^ In contrast to the in vitro performance, the in vivo T cell activation capacity of the NR NVs after intravenous injection outperformed the stimuli-ROS-and/or pH-responsive counterparts, which is likely attributed to the relative in vivo stability of the NVs. In vitro conditions often do not fully replicate factors present in the in vivo setting such as the complex protein and cellular interactions, physiological barriers, and the specific microenvironment of the targeted tissue and in circulation. Follow-up research specifically dedicated to determining in vivo release kinetics of different molecular cargo types and localization thereof and correlation with vaccine efficacy is therefore warranted. Together, our study emphasizes the applicability of polymersome platforms for vaccine applications and highlights the importance of in vivo validation of in vitro data obtained with responsive NVs.

## Supporting Information Available

Table S1: molecular characteristics of the block copolymers as determined by SEC and ^1^H NMR and Table S2: structural features of the manufactured nanovaccines as determined by light scattering measurements and cargo contents (PDF)
