# Supplementary material for: pH and ROS Responsiveness of Polymersome Nanovaccines for Antigen and Adjuvant Codelivery: An In Vitro and In Vivo Comparison
Source: Biomacromolecules. 2024 Jan 18;25(3):1749–58. doi: 10.1021/acs.biomac.3c01235 (PMC10934262; doi:10.1021/acs.biomac.3c01235)
Supplement: Supplementary file 1 — bm3c01235_si_001.pdf [file bm3c01235_si_001.pdf]

Supporting Information

pH-and-ROS-Responsiveness of Polymersome Nanovaccines for Antigen and Adjuvant Co-delivery: An In Vitro and In Vivo Comparison

Eliézer Jäger,[a,b]* Olga Ilina,[b] Yusuf Dölen,[b] Michael Valente,[b] Eric A.W. van Dinther,[b] Alessandro Jäger,[a] Carl G. Figdor[b,c] and Martijn Verdoes[b,c]*

[a]Dr. E. Jäger, Dr. A. Jäger
Institute of Macromolecular Chemistry, Academy of Sciences of the Czech Republic, Heyrovsky Sq. 2, 162 06 Prague, Czech Republic

[b]Dr. E. Jäger, Dr. O. Ilina, Dr. Y. Dolen, Dr. M. Valente, Prof. C.G. Figdor, Dr. M. Verdoes
Department of Medical BioSciences, Radboud University Medical Center, Geert Grooteplein Zuid 28, 6525 GA Nijmegen, The Netherlands.

[c]Prof. C.G. Figdor, Dr. M. Verdoes
Institute for Chemical Immunology, Geert Grooteplein Zuid 28, 6525 GA Nijmegen, The Netherlands.

Email: **jager@imc.cas.cz**; **martijn.Verdoes@radboudumc.nl**

Table S1. Molecular characteristics of the block copolymers used for the manufactured of the nanovaccines as determined by SEC and ^{1}H NMR.

Entry	Block name	M_n ^{1}H NMR (g·mol)$^{-1}$	M_n SEC (g·mol)$^{-1}$	$Đ$ (M_w/M_n)^{c}	ϕ^{d}
$PHPMA_{25}$-*b*-NR_{33}	NR block	13 225^{a}	16 860^{c}	1.10	0.26
$PHPMA_{25}$-*b*-MRE_{30}	MRE block	16 060^{a}	12 300^{c}	1.37	0.22
$PHPMA_{25}$-*b*-MRI_{26}	MRI block	14 760^{a}	14 150^{c}	1.19	0.24
$PHPMA_{37}$-*b*-ROS_{42}	ROS block	18 000^{a}	21 500^{c}	1.13	0.29
$PHPMA_{35}$-*b*-$PDPA_{75}$	pH block	20 990^{b}	22 000^{c}	1.05	0.24

^{a}Determined by ^{1}H NMR in DMF. ^{b}Determined by ^{1}H NMR in D_2O/DCl. ^{c}Determined by SEC in DMF using poly(methylmethacrylate) as the standard. ^{d}Weight fraction of the hydrophilic block based on ^{1}H NMR data.

Table S2. Structural features of the manufactured nanovaccines as determined by light scattering measurements and cargo contents.

Entry	R_H (nm)^{a}	PDI^{a}	R_G (nm)^{b}	R_G/R_H^{b}	ζ (mV)^{c}	Ovalbumine (μg·mL^{-1})^{d}	α-Galcer (ng·mL^{-1})^{e}	LE Ova/α-Galcer (%)
NR-NVs	62.0	0.119	67.0	1.08	- 5.6	6.7	43.0	13.4/4.3
MRE-NVs	60.0	0.116	62.0	1.03	- 6.1	6.8	50.5	13.6/5.0
MRI-NVs	67.0	0.115	70.0	1.04	-2.0	7.8	46.6	15.6/4.7
ROS-NVs	61.0	0.152	63	1.03	-8.0	8.4	58.3	16.8/5.8
pH-NVs	56.0	0.172	61.0	1.08	-7.9	7.6	44.6	15.2/4.5
PLGA	102.0	0.100	-	-	-7.0	4.2*	310*	4.2/31

^{a} Dynamic light scattering. ^{b}Static light scattering. ^{c}Electrophoretic light scattering. ^{d}Determined by Coomassie Plus Protein Assay Reagent. ^{e}Determined by HPLC/CAD. *Content in 0.1 mg/mL of PLGA Nanovaccines.
